# Comparing online travel review platforms as destination image information agents

**DOI:** 10.1007/s40558-021-00201-w

**Published:** 2021-04-22

**Authors:** Xinxin Guo, Juho Pesonen, Raija Komppula

**Affiliations:** Centre for Tourism Studies, Business School, University of Eastern Finland, Yliopistokatu 1, P.O. Box 111, 80101 Joensuu, Finland

**Keywords:** Online travel reviews (OTRs), Content analysis, Destination image, Destination attributes, China

## Abstract

Online travel reviews have been extensively used as an important data source in tourism research. Typically, data for online travel review research is collected only from one platform. However, drawing definite conclusions based on single platform analyses may thus produce biases and lead to erroneous conclusions and decisions. Therefore, this research verifies whether or not there are discrepancies and commonalities between different travel review platforms. In this study, five native Chinese travel review platforms were selected: Ctrip; Qyer; Mafengwo; Tuniu; and Qunar. Using a mixed content analysis method, the destination image of Finland was extracted from 10,197 travel reviews in Simplified Chinese as the destination image is a popular topic in online review research. Results show Finland’s destination image representation varies between Chinese travel review platforms. This discrepancy is especially prominent in the dimension of functional and mixed functional-psychological destination attributes. Significant theoretical contributions and managerial implications for the analysis of online travel reviews and destination image research are discussed.

**Supplementary Information:**

The online version contains supplementary material available at 10.1007/s40558-021-00201-w.

## Introduction

Today, online travel reviews (OTRs) have a huge influence on the tourist decision-making process, because they are often used when tourists compare various options and make travel-related decisions. OTRs are also an indicator of a destination’s post-visit destination image (DI) because tourists write reviews of their experiences based on the image they have after the trip (González-Rodríguez et al. 2016; Park et al. 2007). OTRs are therefore gaining increasing attention in tourism research and destination marketing. Meanwhile, DI is increasingly analyzed using online textual data instead of other data collection methods such as interviews (Lu and Stepchenkova 2015). New analysis methods based on big data allow us to gain in-depth knowledge from this vast social media data ocean (Fazzolari and Petrocchi 2018).

Earlier studies involving OTRs have relied on a single data source (Xiang et al. 2017). In using a single data source for OTR research, researchers ignore platform-specific biases such as differences in platform design, user base, platform-specific behavior, and storage strategy (Pfeffer 2014). Using a single platform is also a potential source of sampling bias that potentially complicates the interpretation of the research findings (Tufekci 2014). This study aims to explore whether or not platform-specific biases in OTRs should be accounted for in tourism research and practice, and if so how. Moreover, earlier studies have mainly used statistical analyses, natural language processing techniques, or algorithms to explore the length of the review, frequently words, topics, and review sentiment (Xiang et al. 2017; Zhang and Cole 2016), or have analyzed the functional features of different websites (Pai et al. 2014).

Additionally, research on OTR platforms in tourism studies is still based largely on the Western context (Xiang et al. 2017). With the rapid growth of Chinese outbound tourists in recent years, scholars are increasingly focusing on China and other Asian countries. The exploration of Chinese social media platforms has become an important research venue (Sotiriadis and Sotiriadis 2017). However, cultural and language barriers mean research on Chinese OTR platforms is rarely published in English. Data from OTR platforms may, therefore, provide a new approach to destination image research among Chinese tourists.

To address these gaps in the previous research, this study makes an in-depth comparison of various native Chinese OTR platforms to identify their potential differences and universal attributes. The differences are analyzed by comparing the DI between platforms as the DI concept is an important topic in OTR analysis (Marine-Roig and Ferrer-Rosell 2018). Additionally, the aim was to explore the reasons for discrepancies and commonalities in the representation of the DI. An instrumental case study approach (Mills et al. 2013) was used in this study as we are interested in the differences of online platforms in representing DI instead of the case itself. Since the research team is familiar with Finland and tourism in the country, it was chosen as the case destination. Finland is also a relatively new destination for Chinese tourists, but growing fast before COVID-19 pandemic. This development aspect makes Finland an interesting case to study the phenomenon. However, we acknowledge that the context is secondary for this research compared to the phenomenon itself and the destination could have been virtually any another destination. The data was collected from five Chinese OTR platforms and analyzed using a mixed content analysis approach focusing on data referring to Finland as a tourist destination. A qualitative content analysis was used to formulate a DI coding manual (for use in the analysis) from part of the samples. A quantitative content analysis was then conducted to objectively extract Finland’s DI from the other OTRs’ data, based on the coding manual.

The structure of the paper is as follows: Chapter 2 presents previous social media studies of OTR platforms. Chapter 3 introduces the theoretical background of the DI framework. The methodology and results are presented in Chapters 4 and 5. A theoretical discussion and practical implications based on the results are presented in Chapter 6. The final chapter includes a conclusion, a discussion of the study’s limitations, and suggestions for future study.

## Social media analytics on online travel review platforms

In 2018, the number of Chinese outbound tourists exceeded 149 million (iResearch 2019). The increasing outbound travel has also led to the increasing use of online travel review platforms in China. With the development of information technologies, China’s tourism information services now cover the pre-travel, on-travel, and post-travel processes (Pan et al. 2019). OTR platforms are especially prominent: 51.4% of outbound tourists obtain travel recommendations and information from Chinese OTR platforms (iResearch 2019). Besides, 71.6% of Chinese outbound tourists share travel experiences on Chinese social media, and 39.9% of tourists share travel experiences on OTR platforms (iResearch 2019). All the evidence indicates that OTR platforms are very important in any attempt to understand outbound Chinese tourists.

There is a vast amount of online information on OTR platforms, commonly known as “big data”. When researchers conduct DI studies based on shared online travel experiences, OTRs are regarded as a form of electronic word-of-mouth communication (eWOM) (Marine-Roig 2017). Although online reviews may be seen as unsolicited and unbiased online information that reflects the realistic tourist perception of the destination (Marine-Roig 2017), the OTR content given by different tourist segments has different focuses (Van der Zee and Bertocchi 2018). Nowadays, the application of OTRs in tourism research has received increasing attention, and researchers usually collect data from a single Western OTR platform, especially TripAdvisor, Yelp, or Expedia (Xiang et al. 2017).

Many researchers have adopted a single OTR platform approach in tourism studies (Xiang et al. 2017). The platform-specific biases of different OTR platforms mean that multi-platform data sources may be more valid in researching tourism phenomena. These biases are not only reflected in the platform design itself (Pai et al. 2014), but in the posting behavior of tourists and managers (Pfeffer 2014). The research already demonstrates that the major Western OTR platforms differ regarding the cost of reviewing (Chevalier et al. 2018; Zhuang et al. 2018), the review content posting behavior in terms of the number of reviews, the review length, customer preference, and sentiment, for example (Proserpio and Zervas 2017; Wang and Chaudhry 2018; Xiang et al. 2017).

Researchers are thus well aware of the differences between major Western OTR platforms. However, although we often use OTR platforms for destination image analytics (Marine-Roig and Ferrer-Rosell 2018), how the OTR platform itself affects DI analytics remains unknown. It is imperative to understand how the DI differs between different platforms or whether there is a difference at all. We, therefore, compare Chinese OTR platforms and analyze the results of online DI analytics from major Chinese OTR platforms.

## The framework of the destination image

The usual definition of DI is the sum of a person’s beliefs, ideas and impressions of a destination (Crompton 1979). It is formed in a process in which personal, sociocultural, and information technology factors (Beerli and Martin 2004; Josiassen et al. 2015; Kislali et al. 2016; San Martín and Del Bosque 2008), as well as stimulus factors (e.g., information sources, previous experience of the destination) affect the formation of the image (Gartner 1993). According to Gartner (1993), destination information can be regarded as a continuum of various image formation agents, ranging from traditional forms of the induced agent to autonomous and organic image agents. “Induced agents” refers to the information provided by commercial destination actors representing the supply-side view of DI as the projected image (Mak 2017). “Autonomous image agents” refers to information sources which are not under the control of the destination organizations, referring to news, movies, and documentaries, for example (Gartner 1993). “Organic image agents” refers to information sources based on a visit to the area (Gartner 1993).

With the development of information technology, induced and organic image formation agents are not necessarily mutually exclusive but may complement each other (Selby and Morgan 1996). One view is that the Internet can be seen as an induced information agent in the image formation process (Beerli and Martin 2004). The opposing view is that the previous point is outdated in the modern online environment, and the different online travel platforms (such as official tourism websites, travel blog platforms, or travel review platforms) on the Internet can be classified as induced, autonomous, or organic information agents (Llodrà-Riera et al. 2015). Online destination information can, therefore, be regarded as an agent of induced or organic image formation, both of which play a significant role in the image formation process (Llodrà-Riera et al. 2015). Besides, when tourists obtain destination information from different online travel platforms, there may be a discrepancy between the destination images based on official tourism website content (induced), travel blog platform content (autonomous), and travel review platform content (organic) (Mak 2017; Marine-Roig and Ferrer-Rosell 2018). Perceptions of official tourism website content (induced) and travel blog platform content (autonomous) differ less from each other (Marine-Roig and Ferrer-Rosell 2018).

Due to the OTRs’ source credibility and information quality, travel review platforms as organic information agents are more unbiased and trustworthy than the induced information agents of official tourism websites (Filieri et al. 2015). The assessment of DI formation based on OTR data is, therefore, becoming increasingly popular. In particular, understanding DI based on different OTR platform content may assist in exploring whether there is a DI discrepancy between different organic information agents. Mak (2017) used the term online destination image to depict “the online representation of the collective beliefs, knowledge, ideas, feelings and overall impressions of a destination.”

There are two main approaches to defining DI construction. One considers DI as a multidimensional construct with two main components: the cognitive image and the affective image of destinations (Baloglu and McCleary 1999). These two images respectively represent a tourist’s knowledge of the destination and their emotions based on their destination knowledge (Baloglu and McCleary 1999; Gartner 1993). The other most-cited construction is considering DI as a person’s overall evaluation of the destination, which includes attribute-based and holistic components (Echtner and Ritchie 1991, 1993). Each component can be further subdivided into functional-psychological; or common-unique characteristics (Echtner and Ritchie 1991, 1993). The attribute-holistic continuum illustrates whether the representation of DI is from the perspective of an individual attribute or a holistic aggregate. The functional-psychological continuum refers to functional (directly observable or measurable) or psychological (less tangible, difficult to measure) attributes. The common-unique continuum also refers to common characteristics, attributes, and impressions according to which destinations are commonly compared, or it refers to unique or destination-specific features (Echtner and Ritchie 1991, 1993). By introducing a three-dimensional DI framework, Echtner and Ritchie (1993) developed a 35-item destination attribute scale, ranging from more functional attributes (including tourist sites/activities, national parks, and historic sites) and mixed destination attributes (including crowdedness, cleanliness, and political stability) to more psychological destination attributes (including hospitality, place atmosphere, and reputation).

Subsequently, some studies have proposed various scales to determine the destination attributes and measure the DI (Beerli and Martin 2004; Choi et al. 2007; Enright and Newton 2005; Gallarza et al. 2002; Marine-Roig 2017; Rodrigues et al. 2015; Vinyals-Mirabent 2019). In this study, we have combined Echtner and Ritchie’s (1993) functional-psychological attribute scales and Beerli and Martín’s (2004) attribute classification as an adapted framework (see Appendix 1) for data analysis. Echtner and Ritchie’s (1993) study placed 35-item destination attributes into a functional-psychological scale, which does not cover all the universal attributes in the destination. Therefore, another often cited destination attribute study by Beerli and Martín (2004) was applied for the adapted framework. Beerli and Martín’s (2004) study classified destination attributes into nine dimensions, but they did not distinguish the functional or psychological features of these attributes. For this reason we developed an adapted attribute framework which combines the advantages of Echtner and Ritchie’s (1993) and Beerli and Martín’s (2004) studies.

In order to build the adapted attribute framework, the first step was to place Echtner and Ritchie’s (1993) 35 identified destination attributes into Beerli and Martín’s (2004) destination attribute classifications. Then, according to the functional-psychological definition of the attribute given in Echtner and Ritchie’s (1993) study, the functional and psychological feature of the attribute classification were determined. For example, tourist sites, tourist activities, sports activities, national parks, and tourist entertainment were regarded as functional destination attributes in Echtner and Ritchie’s study (1993). In Beerli and Martín’s (2004) study, these attributes were classified as a tourism leisure dimension. Therefore, the tourism leisure dimension was considered a functional attribute after some research group discussion. Moreover, in the tourism leisure dimension, architecture and buildings, which were not covered in Echtner and Ritchie’s (1993) study but were identified in Beerli and Martín’s (2004) study, were also considered as a functional destination attribute. The adapted DI framework comprehensively illustrates the destination attributes from continuous functional to psychological characteristics in nine dimensions. The tourism leisure and recreation, natural resources, and tourism infrastructure dimensions are more related to the functional level. On the other hand, the dimensions of culture, history, art, general infrastructure, and natural environment belong to the mixed functional-psychological level. The abstract psychological attributes include politics and economics, the social environment, and the atmosphere of the place in question.

As socio-demographic and sociocultural factors (Beerli and Martin 2004; Josiassen et al. 2015; Kislali et al. 2016; San Martín and Del Bosque 2008) play an important role in the image formation process. It can be assumed that tourists with different cultural backgrounds may perceive the same destination attribute differently (Nakayama and Wan 2019). As most of the academic research on destination image analytics has been conducted Using western platforms, a short review of the literature focusing on Chinese tourists’ perceived images of Western destinations may highlight the dimensions of the image the Chinese tourists’ highlight. Chinese tourists retain different preferences for domestic and Western destinations (Li and Stepchenkova 2012; Wang and Hsu 2010). In domestic travel, the service quality attribute is the most important factor in shaping the DI (Wang and Hsu 2010). However, most Chinese tourists visiting Western countries share travel experiences concerning natural resources and local cultures (Huang and Gross 2010; Li and Stepchenkova 2012; Sun et al., 2015). Chinese tourists are also willing to discuss political and economic issues affecting Western destinations (Li and Stepchenkova 2012). To confirm judgements about Western destinations, Chinese outbound tourists tend to compare differences between a Western destination’s social systems and China’s (Huang and Gross 2010). Additionally, Chinese cultural norms play an important role in the process of perception formation and the interpretation of Western destinations (Sun et al. 2015). These cultural norms include the desire for harmony and respect for the authorities. The different cultural backgrounds of Chinese and Western tourists mean there may be significant differences in perceptions of the same destination (Tang et al. 2009). According to Kim and Morrison (2005), Chinese outbound tourists are more likely to change their perception of destinations in a short period than Western tourists.

## Methodology

Adopting the mixed qualitative and quantitative content analysis approach, this study compared the representation of the image of Finland in different Chinese OTRs, to interpret the commonalities and discrepancies between various platforms. China has become one of the largest source markets in international tourism (UNWTO 2018), and this growth has also been witnessed in Finland. Between 2011 and 2018, the number of Chinese tourists visiting Finland increased by 323% (Statistics Finland 2019). In 2012, the Finnish national tourism office (VisitFinland) and the Finnish airline company Finnair established digital marketing strategies on Weibo, in China. Although the data reveals that the Nordic countries have great potential in the Chinese market, gaps and deficiencies remain in DI research in the Nordic countries (Andersen et al. 2018). Today, China has the largest market of Internet users, accounting for 21% of the worldwide total (Meeker 2019). With an increasing number of Chinese tourists sharing travel experiences online, the massive amount of information they generate provides researchers with a way of studying the DI of a European destination from the perspective of Chinese tourists. Even though the study uses Finland and China as examples, the results can be generalized to other market combinations.

**Fig. 1:**
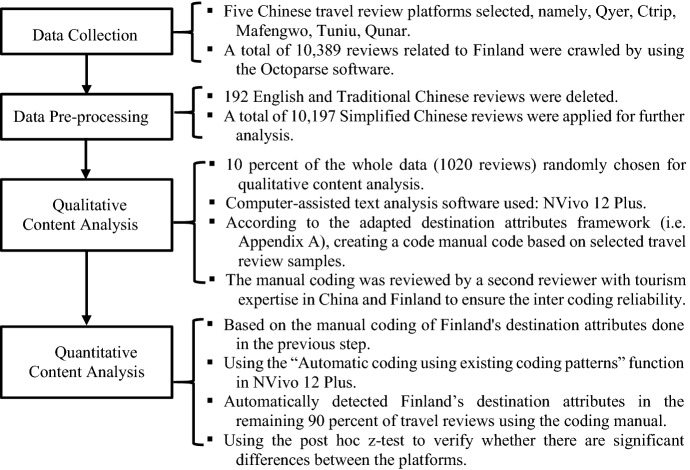
The flowchart of the research process

In China, travel websites with review functions can be classified in two main categories: travel vertical platforms, such as Mafengwo, Qyer; and online tour agents (OTA), such as Ctrip, Tuniu, and Qunar (Graff and Parulis-Cook 2019, p. 53). Vertical travel websites rely heavily on user-created content, and provide tourists with generated travel information and related travel-specific services (Graff and Parulis-Cook 2019; Kizmaz 2018). Chinese OTA websites provide many travel-related services including visa arrangements, tax refunds, and financial services, as well as travel information. Many Chinese OTA websites now also have a review function for users to share their comments about destinations. Before entering the detailed introduction of the research method, a flowchart (Fig. 1) summarizing the key information of the research process is shown below.

The flowchart shows the four main parts of the research process, namely data collection, data pre-processing, qualitative content analysis, and quantitative content analysis. The following chapters will introduce each of these steps in detail.

### Data collection and pre-processing

**Fig. 2:**
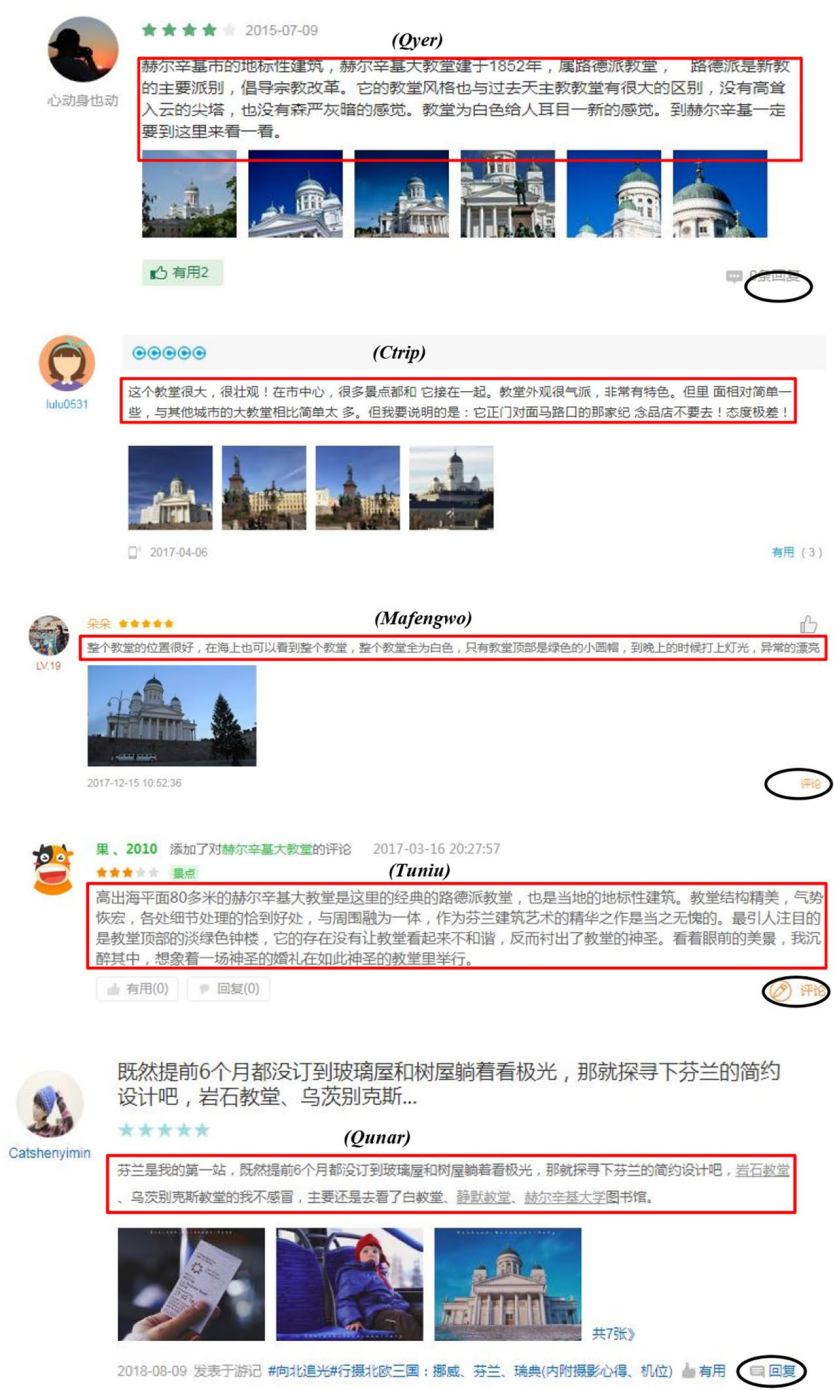
The format of the OTRs on Qyer, Ctrip, Mafengwo, Tuniu, and Qunar

Baidu is the largest online search engine in China. Its information center (http://site.baidu.com/) displays 23 popular Chinese tourism websites. Manually typing “芬兰” (Finland) into the search engine of each tourism information website resulted in six websites with Chinese OTRs for Finland. These OTRs were on Qyer, Ctrip, Mafengwo, Tuniu, Qunar, and Maotuying (the Chinese version of TripAdvisor). On Maotuying, the OTRs in Chinese are translated from other languages. This platform was therefore excluded from the study. Table 1 displays background information about these five platforms. Figure 2 shows the format of the OTRs on different platforms. OTRs are basically comprised of four components: linguistic features; semantic features; sentiment; and reviewer information (Xiang et al. 2017). The differences in travel platform design mean that not all these features can be found on each platform (Xiang et al. 2017). For example, except for Tuniu, tourists can attach photos to OTRs on the other four platforms. On Qyer, Mafengwo, Tuniu, and Qunar tourists can comment on others’ OTRs. However, all the platforms contain basic review features (textual review content, ratings, and the release time) and reviewer’s information (nickname, profile photo).

**Table 1:** Basic information on the selected Chinese OTR platforms

Name	Qyer	Ctrip	Qunar	Mafengwo	Tuniu
Website	qyer.com	ctrip.com	qunar.com	mafengwo.cn	tuniu.com
Type of travel website	Vertical	OTA	OTA	Vertical	OTA
Founded (year)	2004	1999	2005	2006	2006
Total users	100 million (2019)	300 million (2018)	218 million (2019)	120 million (2018)	Missing data
Active APP users (monthly)	Missing data	61.2 million (August 2018)	36.6 million (August 2018)	9.8 million (August 2018)	7.8 million (August 2018)
Target user group	Low- and middle-income white-collar workers and college students	Middle- and high-income white-collar workers	Low- and middle-income white-collar workers	Middle- and high-income white-collar workers	Middle- and high-income white-collar workers
Product positioning	Self-guided travel	Business travel	Self-guided travel	Self-guided travel	Group travel
Market positioning	The largest Chinese service platform to assist Chinese tourists in their personal experience of outbound travel	A comprehensive tourism service platform with the largest market share in China, offering all kinds of travel services	One-stop solutions for meals, accommodation, shopping, entertainment, and other reservation services	The leading Chinese service platform for self-guided tours, offering personalized travel services	With tourist routes as the core service, providing leisure tourism services

Information from Baidu’s official website (Baidu 2019) and (Smith 2019)

In Fig. 2, the red box indicates the content of the OTRs. The black circle indicates the reply function.

Data collection was conducted using a web crawler, Octoparse, which was used to extract the required data information from the hypertext markup language on the travel review webpages. In this study, we collected only the textual review content, release date, and reviewer’s nickname. The collection process took place between early October and the end of December 2018. A total of 10,389 OTRs related to Finland were crawled by the setting crawling process. The textual OTR content includes descriptions of attractions, hotels, restaurants, entertainment activities, and others. Furthermore, only Simplified Chinese OTRs were considered in this study. After deleting 192 English and Traditional Chinese OTRs, a total of 10,197 Simplified Chinese OTRs were applied for further analysis. As Table 2 shows, Qyer had the largest number (3570) of OTRs, followed by Ctrip and Qunar. Mafengwo and Tuniu had a nearly equal number of OTRs and are the smallest platforms.

**Table 2:** Summary of OTR dataset

OTR platform	Number of OTRs	Percentage of total (%)
Qyer	3570	35.01
Ctrip	2394	23.48
Qunar	1738	17.04
Mafengwo	1271	12.46
Tuniu	1224	12.00
Total	10,197	100.00

### Data analysis

The content analysis approach is commonly adopted to analyze textual messages (Stepchenkova and Mills 2010). It can be used to compress many words into categories based on explicit coding rules (Harwood and Garry 2004). Most of the existing literature used either the qualitative or quantitative content analyses to study the perceived destination from OTRs, and the quantitative approach seems to be the mainstream choice (Marine-Roig and Ferrer-Rosell 2018; Qi et al. 2018; Zhang and Cole 2016). Applying the computerized quantitative content analysis approach to OTR-based image studies includes two basic steps, data pre-processing and attribute identification (Marine-Roig and Clavé 2016; Xiang et al. 2017). Data pre-processing generally involves some operations, including tokenization (means breaking the review text into words, phrases, or other meaningful elements), and removing stop words (e.g. a, the, so, or other words do not contribute to the meanings of the text) (Xiang et al. 2017). Attribute identification in a quantitative content analysis aims to detect the frequency, density and weight of keywords or key phrases in the content by computer program, and then aggregate keywords or key phrases into destination attribute categories (Marine-Roig and Clavé 2016). Because a quantitative content analysis often focuses on searching for keywords, adopting a quantitative computerized approach alone often results in ignoring valuable contextual information embedded in the OTR data (Stepchenkova et al. 2009; Zhang and Cole 2016).

In contrast to the quantitative content analysis approach, the qualitative content analysis approach is the subjective interpretation of textual content, and used to manually extract the DI from a small number of tourists’ narrative descriptions (Sun et al. 2015; Tegegne et al. 2018). Using the qualitative content analysis approach could extract the valuable contextual information embedded in the textual content. The systematic classification process of encoding the destination attributes and identifying attribute categories is the core of the qualitative content analysis approach in DI studies (Lian and Yu 2017). In addition, inter-coder reliability must be carefully considered, which means that different coders need to produce the same encoding results in the same way (Lian and Yu 2017). Although a qualitative content analysis focuses on valuable contextual information embedded in the text content, manual coding is quite time-consuming to apply for large-scale text analysis. In order to solve the two problems of extracting valuable information embedded in review content, and processing large amounts of OTR data, thus, a novel approach combining both qualitative and quantitative methods was applied in this study.

The qualitative content analysis in this study was conducted first to identify Finland’s destination attributes and build up a coding manual. In this process, the coding of the destination attributes and categorization followed the adapted attribute framework from previous studies (see Appendix 1). Basically, the adapted attribute framework ensured the validity of encoding destination attributes and identifying attribute categories. Therefore, two coders randomly chose 10% of the travel reviews to formulate a coding manual of Finland’s destination attributes. All data coding was conducted on Chinese-language texts using the computer-assisted text analysis software NVivo 12 Plus. The data reached a saturation point when adding additional OTRs failed to reveal novel aspects or issues (Papathanassis and Knolle 2011). The coding manual was built by using the following steps: (a) an OTR was read carefully and destination attribute were identified based on the context of review content, (b) the identified attribute was verified in the adapted attribute framework, (c) the code was confirmed if the identified attribute existed in the framework, (d) if the identified attribute did not exist in the framework, the coders discussed and decided on the attribute code and its classification. Furthermore, in order to ensure the reliability of the coding manual, a second reviewer with tourism expertise in China and Finland was asked to review the codes.

In the process of formulating the coding manual, several operations were performed on the selected reviews. First, the coders made efforts to unify the spelling of the names of attractions on different review platforms. For example, the description of Kamppi Chapel and the Silent Church pointed to the same attraction, which was coded as “Kamppi Chapel” under the destination attribute code “churches”. Second, universal terms of destination attributes were applied in the cases that Chinese tourists mentioned general infrastructure without giving a specific name. For instance, Chinese tourists gave descriptions of Finland’s libraries without referring to a certain place, thus the general terms “libraries” were applied to these descriptions.

Based on the coding manual from the qualitative content analysis, a computerized quantitative content analysis was applied to the remaining data by using the “automatic coding using existing coding patterns” function in NVivo 12 Plus. The premise of using pattern-based auto-coding is that the coder needs to manually code part of the material. When using the identified codes for automatic coding, NVivo compares each text part (e.g., a sentence or paragraph) with the review content already coded into the destination attribute. If the content of the text paragraph is similar in wording to the content already coded for the destination attribute, the text paragraph will be coded for that identified attribute. In doing so, the quantitative content analysis results can then reveal Finland’s image on the various Chinese OTR platforms. This study further used the post hoc *z* test to verify whether the differences between Finland’s image on different platforms are significant. The chi-square post hoc z-test using adjusted residuals is applied to detect differences between groups data (Zhang et al. 2017). The premises of using the z-test are that the variance is known and the sample size is large enough (sample size ≥), as is in this case (Table 4). The test shows the cells in the chi-square table that have significantly lower or higher adjusted residuals on the 95% confidence interval.

## Results of the destination image analysis

### Qualitative analysis results for Finland’s destination image categories

Compared with the adapted DI framework (see Table 3), the qualitative content analysis results proved that Finland’s Chinese OTR data-based DI covered all nine destination attribute dimensions, from the functional to the psychological levels. However, within every attribute dimension, while some universal destination attributes did not appear, other new destination attributes were identified in the data.

**Table 3:** Finland’s destination attributes identified in Chinese OTRs

Finland’s attribute dimension		Destination attributes (no. of coding)
FUNCTIONAL	Tourism leisure and recreation	**City parks (4)**; international events (3); national parks (4); performance events (3); playgrounds (1); sports activities (4); sports stadiums (2); theme parks (3); tourist entertainment activities (9); zoos and botanical gardens (3)
	Natural resources	Air (1); natural ecosystems (5); natural phenomenon (1); seasons (4); variety and uniqueness of flora and fauna (2); weather (6); beaches(0); a wealth of countryside (0)
	Tourism infrastructure	Bars (3); destination accessibility (5); hotels (5); resorts (3); restaurants (3); tourist centers (3); online information and services (3); **payment methods (3)**
	Culture, history, and art	Ancient cities (1); art attractions (8); castles and fortresses (4); churches (9); festivals (4); gastronomy (7); handicrafts (2); museums and monuments (22); ruins (1)
	General infrastructure	Bridges (1); **educational facilities (2)**; financial service facilities (1); **government places (3)**; **national industry (2)**; public transportation facilities (7); shopping and market facilities (5); streets and roads (3); development of health services(0); development of telecommunications(0)
	Natural environment	Beauty of the scenery (2); crowded and fewer tourists (2); hygiene situation (2); noise pollution (1); traffic congestion (0)
	Political and economic factors	Political environment (1); political stability (1); prices (6); safety (3); degree of urbanization (0); economic development (0)
	Social environment	**Ethnic origins (2)**; hospitality of local residents (2); language barriers (1); local lifestyle (3); local values (3); the underprivileged and poverty (1)
PSYCHOLOGICAL	Atmosphere of place	**Ancient and historic atmosphere (2)**; **artistic atmosphere (2)**; attractive or interesting (2); boring (6); **commercialized (2)**; **desolate or depressing (5)**; exotic (5); family-oriented destination (1); fun or enjoyable (6); **harmonious (3)**; **majestic (4)**; place with a famous reputation (1); **fairytale or magical (3)**; relaxing (4); romantic (1); **solemn (2)**; worthy or meaningful (3); opportunity for adventure(0); mystic vs. prosaic (0); luxurious vs. impoverished (0); fashionable vs. outdated(0)

In the table, the bold destination attributes indicate new items compared to the adapted frameworks (see Appendix 1). If the value in parentheses is zero, the attribute does not appear in the Finnish destination image

The following paragraphs demonstrate some of the aspects identified with some quotations from the source data to illustrate the points.

At the functional level, attributes identified in the adapted frameworks such as beaches and the richness of the countryside did not appear in the Chinese OTRs. However, other attributes were identified in the selected OTR samples, for example, the new attributes of city parks (Quotation 1) and payment methods (Quotation 2) were identified at the functional level. This can be seen in the following quotations:

- *Quotation 1* “*Sibelius Park is located about 1.5 km northwest of Temppeliaukio Church. It was built to commemorate the great Finnish musician Sibelius. The park is full of flowers and green grass, …”—Reviewer (Case number: 5143) from Ctrip.*
- *Quotation 2 “… At the terminal, we bought a round-trip ticket for 5 euros at the self-service ticket vending machine. It seems that only cash is accepted, and no credit card was accepted. ….”—Reviewer (Case number: 3474) from Qyer.*

At the mixed functional-psychological level, attributes concerning the development of health services and telecommunications and traffic congestion which were in the adapted framework were missing. However, a few new attributes, such as educational facilities (Quotation 3), and national industry (Quotation 4) were identified at the mixed functional-psychological level. The following quotations illustrate these aspects.

- *Quotation 3: “The informatization of Finnish libraries is very good. Finns can borrow materials from the public libraries… and return books to another library which is near their home, …”—Reviewer (Case number: 9583) from Mafengwo.*
- *Quotation 4: “… Known for its technology-intensive industries, it has become a leading technology center in the Nordic region, where Nokia's headquarters is located.”—Reviewer (Case number: 5700) from Qyer.*

At the psychological level, aspects from the adapted framework including the degree of urbanization, economic development, the opportunity for adventure, the mystic vs prosaic aspect, the luxurious vs impoverished nature of the destination, or fashionable vs outdated elements could not be discerned. This did not mean that Chinese tourists were not aware of Finnish destination attributes at the psychological level. On the contrary, Chinese outbound tourists seem to have an abundant and unique psychological perception of Finland, especially concerning the atmospheric dimensions. The following quotations (Quotation 5, 6) emphasize the “harmonious” atmosphere.

- *Quotation 5 “…there are a lot of people of different skin color sitting on the steps, sunbathing and chatting, and the whole atmosphere is very harmonious and enjoyable.”—Reviewer (Case number: 9172) from Qyer*
- *Quotation 6 “There are also food stalls in the market, there are fruit sellers and handicraft sellers. This free market is across the road from* the *presidential palace and other government buildings—what a great harmonious society scene!”—Reviewer (Case number: 10030) from Tuniu.*

Table 3 shows Finland’s destination attributes identified in Chinese OTRs. As can be seen most of the attributes appeared at the psychological level including attributes concerning ethnic origins, an ancient and historic atmosphere, an artistic atmosphere, as well as desolate, depressing, harmonious, majestic, fairytale, magical and solemn attributes.

### Quantitative analysis results of Finland’s image on five review platforms

Based on the coding manual created in the qualitative analysis phase, a quantitative content analysis for the remaining 90% of OTR data was conducted. The results presented in Table 4 show that Chinese tourists visiting Finland generally perceived Finland as a leisure destination with various cultural, historic, and artistic elements. The culture, history, and art dimensions had the largest amount of coding references, accounting for 20.32% of the total, followed by the tourism leisure and recreation dimensions, accounting for 19.51%. These two attribute dimensions accounted for a large share of the Finland’s DI at the functional level. The largest dimension at the psychological level was the place atmosphere, with a 19.25% share of mentions. Furthermore, the shares of the dimensions encompassing Finnish natural resources, natural environment, political and economic, and social environment were 5.52, 5.69, 3.53, and 2.35%. The proportions of the latter three dimensions were much lower than the proportions of the first three dimensions.

**Table 4:**
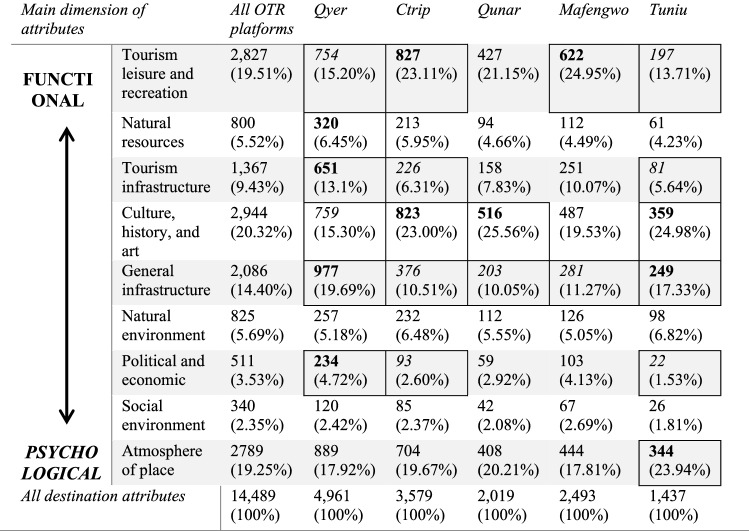
Finland’s destination image (amount of attributes references) on different OTR platforms

Bolded figures signify statistically higher actual count than expected count and italic figures signify statistically lower actual count than expected count (Sig. level 0.05, number of tests = 45, adjusted sig. level 0.00111, *z* criteria ± 3.261)

**Table 5:** The destination image framework and attribute

Functional destination attributes (Physical, measurable)		
	Tourism leisure, recreation	Theme parks and activities; national parks and wilderness activities; sports facilities and activities; architecture and buildings; tourist entertainment and activities (nightlife, movie, shopping etc.)
	Natural resources	Climate and weather; beaches; wealth of countryside; variety and uniqueness of flora and fauna
	Tourism infrastructure	Accommodation; restaurants; bars, discos and clubs; destination accessibility; facilities for information and tours; network of tourist information
	Culture, history, art	Historic sites, museums, monuments, etc.; festivals, fairs, exhibitions, concerts, etc.; handicrafts; different cuisine/food/drink; folklore; religion; customs and ways of life
	General infrastructure	Development of the transportation system; private and public transportation facilities; development of health services; development of telecommunications; development of commercial infrastructure
	Natural environment	Natural attractions and scenery; attractiveness of cities and towns; cleanliness; crowdedness; air and noise pollution; traffic congestion
	Politics, economics	Political stability; political tendencies; degree of urbanization; economic development; personal safety; costs and price level
	Social environment	Hospitality, friendliness and receptiveness of the local residents; underprivilege and poverty; quality of life and values; language barrier
	Atmosphere of place	Good reputation; children-, adult-, or family-oriented place; opportunity for adventure; opportunity to increase knowledge; fun vs. boring; relaxing vs. stressful; attractive vs. unappealing; familiar vs. exotic; mystic vs. prosaic; luxurious vs. impoverished; fashionable vs. outdated
Psychological destination attributes (abstract)		

Adapted from Echtner and Ritchie (1993) and Beerli and Martín (2004)

As can be seen from Table 4, the results therefore also reveal a discrepancy in Finland’s DI based on different platform OTRs. From the number of destination attribute references, the total number of destination attribute references came to 4961 on Qyer, the largest of the other four platforms, followed by Ctrip, with 3579, Mafengwo, with 2493, Qunar, with 2019, and Tuniu, with 1437 references. Qyer had a greater number of attribute references than the other four platforms concerning the natural resources dimension (320), tourism infrastructure dimension (651), general infrastructure dimension (977), natural environment dimension (257), political and economic dimension (234), social environment dimension (120), and the place atmosphere dimension (889). Ctrip had a greater number of destination attributes references concerning the tourism leisure and recreation dimension (827), and the culture, history, art dimension (823). The remaining three platforms Qunar, Mafengwo and Tuniu did not have the largest number of references for any destination attribute dimension.

The *z* test showed that the differences between the five travel review platforms were significant. Reviews on Qyer discuss natural resources, tourism infrastructure, general infrastructure, and political and economic situation significantly more than what could be expected. However, there is significantly less information about tourism leisure and recreation, and culture, history and art compared to other platforms. These two destination image dimensions are more prominent on Ctrip, as well as tourism infrastructure. However, Ctrip lacks reviews on general infrastructure, as does Qunar and Mafengwo. Tuniu seems to focus the most on culture, history, and art as well as the atmosphere of the place. The results are interesting also in the destination image dimensions. It seems that the most significant differences are in functional dimensions whereas psychological dimensions are relatively similar between different platforms.

## Discussion

There is no doubt that big data has had a major impact on tourism research (Li et al. 2018). Whereas most previous studies have used a quantitative content analysis approach with a single OTR platform (Lalicic et al. 2021; Tseng et al. 2015; Xiang et al. 2017), we explored the differences and universal attributes of various OTR platforms with a mixed qualitative and quantitative content analysis approach. This approach allowed us to extract valuable contextual information embedded in a large amount of OTR data. Additionally, in applying Simplified Chinese OTR content from multiple Chinese-based platforms, this study interpreted Finland’s image in terms of nine destination attribute dimensions of a functional and psychological destination attribute scale. In this study, we also identified statistically significant differences between the review topics on various Chinese OTR platforms. Users of different platforms discuss different issues in their reviews. These differences can have a significant effect on what kind of conclusions are drawn from destination image studies based on OTRs. We also found that Chinese online travel reviews of Finland focus on functional and mixed psychological-functional destination attributes.

### Differences in destination image between OTR platforms

First of all, this study aimed to explore if and how the destination image differed on various OTR platforms (Table 4). The results of this study show that there are indeed many differences between platforms. This is an important observation for researchers as well as tourism managers. OTRs are often analyzed to understand DI (Lalicic et al. 2021; Marine-Roig and Clavé 2016; Marine-Roig and Ferrer-Rosell 2018). The results demonstrated how the conclusions made about DI can differ depending on where the OTR analyzed come from. For example, if only Ctrip or Mafengwo reviews are analyzed the results would show that the Chinese tourists focus on tourism leisure and recreation. However, if only Tuniu reviews were analyzed the results would show that Chinese tourists focus and pay attention mainly to culture, history, and art. Only by including a wide range of sources and data is it possible to form a comprehensive picture of the actual DI.

Previous research has shown that Western OTR platforms (TripAdvisor, Expedia, and Yelp) have discrepancies in their displays of the hotel product, and each platform has its own characteristics (Xiang et al. 2017). According to the results of this study, platform-based specific characteristics in the representation of Finland’s image exist based on different Chinese platforms’ OTR content. Tourism studies, especially DI research, should take into account platform-specific biases, and data collected from multiple OTR platform can better reflect DI compared to individual platforms. These platform-specific characteristics are especially prominent in the functional and mixed functional-psychological dimensions.

Earlier studies have also argued that these platform-specific content characteristics were explained by the different user groups on each OTR platform (Xiang et al. 2017). However, based on the Chinese OTR platforms, it seems that the differences cannot be directly reflected by their target user groups. For example, both Ctrip and Tuniu focus on middle- and high-income tourists, but the platform-specific characteristics in their OTR content still differ. These differences, therefore, seem to be the result of multiple factors, including not only the user group but their online product, marketing, or market positioning. It is critical to understand these differences when conducting social media analytics research for destination image analyses. Where the data is collected matters. Various platforms’ destination image analyses provide different results. Drawing definite conclusions based on single platform analyses can thus produce biases and lead to incorrect conclusions on what the customers thinkg about the destination (Pfeffer 2014).

### OTRs as organic image information agents

Second, this study demonstrates that OTRs, as organic image information agents, can contribute significantly to functional and psychological destination attributes, and are especially prominent at the functional and mixed functional-psychological level. This result is interesting when compared to the earlier literature. Earlier studies have shown that organic image formation agents (e.g., word-of-mouth) affect the perception of psychological destination attributes, whereas induced (advertising) and autonomous (non-promotional) sources contribute to the formation of functional destination attributes (Baloglu 2000; Mak 2017). In this study, the results show that, overall, the functional attributes and mixed functional-psychological attributes had more descriptions than the psychological attributes in Chinese OTRs. This was especially true for the tourism leisure and recreation attribute dimension; the and culture, history, art dimension. The reason for this may be related to the development of information technology. The application of portable communication devices and the popularization of information networks have made it easy for tourists to share all aspects of their travel experience through social media platforms (Huertas and Marine-Roig 2016).

This study also contributes to the tourism literature by revealing the relational nature of DI and its formation by organic image information agents referring to OTR platforms. The results of this study confirm that each destination may have its own destination attribute scale, which is consistent with Beerli and Martín’s (2004) study. Although, Finland was perceived as a leisure destination with various cultural, historic, and artistic elements, and all nine destination attribute dimensions were recognized in travel reviews, some universal destination attributes such as beaches and the wealth of the countryside did not appear and some new attributes such as city parks and payment methods were identified (Table 3). The results expand our knowledge on the topics that tourists pay attention to when they are travelling and what factors can affect the perceived destination image.

Furthermore, according to the results of this study, most Chinese tourists pay less attention to the political attributes in Finland, including safety issues, the political environment, and political stability. However, more than ten years ago, when OTR platforms were rarely used by Chinese outbound tourists, most considered safety one of the most important destination attributes (Kim et al. 2005). The development of information technology may explain this: tourists now have more diverse ways of obtaining destination information through social media, leading to a more comprehensive understanding of destinations and thus minimize their risk perception. Tourists assist each other especially through OTR platforms, which may be beneficial for risk reduction (Jacobsen and Munar 2012).

### Practical implications

Chinese tourists use organic image information agents, OTRs, as influential destination information sources (iResearch 2019). Due to the discrepancy in the representation of DI on different OTR platforms, this study provides at least four implications for all DMOs. Western DMOs could use the knowledge concerning destination image analysis to position themselves in the Chinese market and modify their service design and marketing processes.

First, these Chinese platforms provide not only OTR functions to tourists but also travel products to consumers, including flights, accommodation, and tour guide services. Based on the background of each platform and the DI it represents, DMOs may need to develop suitable product strategies for each OTR platform and different target groups. The most efficient marketing combinations come from marketing and selling the correct tourism service on the correct channel.

Second, DI directly affects the travel intentions of potential tourists (Chaulagain et al. 2019). Today, induced and organic sources may complement each other (Selby and Morgan 1996). DMOs should focus on the image displayed by OTR content and try to increase interactivity with tourists (Huertas and Marine-Roig 2016), especially on opinion leaders’ OTRs. As OTRs become increasingly influential, a DI represented on OTR platforms will increasingly affect the perceived DI of tourists searching for travel-related information (Marine-Roig and Ferrer-Rosell 2018). Indubitably, opinion leaders’ OTRs have a great impact on the DI of potential tourists (Jalilvand 2017). Their OTRs may enhance DI and make the information recipient feel the destination is attractive. Their comments may also weaken the DI and discourage tourists from visiting a destination. Given that most Chinese OTR platforms offer a comment function (see Fig. 1), DMOs could supplement destination information or repair damaged DI through opinion leaders’ OTRs (Chevalier et al. 2018).

Third, DMOs should aim to reduce the gap between tourists’ perceived and the destination’s projected images. Tourists will thus have satisfying experiences and perceive a strong destination brand (Marine-Roig and Ferrer-Rosell 2018). Understanding tourists’ perceived images are crucial here. In reducing the gap between these two kinds of image, DMOs need to adjust marketing strategies, and the entire destination may even need to be developed in tune with the realities of the general infrastructure and social and political environments (Marine-Roig and Ferrer-Rosell 2018).

Fourth, DMOs should pay great attention to the influence of tourists’ cultural backgrounds and demographic characteristics on the perception of the destination. The image formation process is not only affected by destination information sources (induced, autonomous, and organic) but also by sociocultural and socio-demographic characteristics (Josiassen et al. 2015; Nakayama and Wan 2019; San Martín and Del Bosque 2008). Chinese outbound tourists emphasize Chinese cultural norms in the process of image perception and interpretation (Sun et al. 2015). Therefore, Chinese tourists seem to have an abundant and unique psychological perception in the place atmosphere dimension (Table 3), the “harmonious atmosphere” attribute was especially identified in the review content. In addition, most users of China’s OTR platforms are white-collar workers (Table 1), and their average age is around 35 (iResearch 2019). Young Chinese outbound tourists’ emphasize local culture and novel travel experiences (Sparks and Pan 2009), and consequently Chinese tourists have a strong perception of a destination’s culture, history, and art attributes. These findings prove that understanding the characteristics of tourists is of great help to grasp the reasons behind DI formation.

In addition to the contributions this study has for all destinations, this study also provides three insights for Finnish DMOs. First, to improve Chinese tourists’ awareness of Finland, Finnish DMOs should strengthen their cooperation with OTR platforms like Tuniu, Qunar, and Mafengwo, or find Chinese agents to launch Finnish tourism products on them. Of the nine dimensions of DI analyzed, Chinese tourists seem to lack an awareness of the Finnish social and political environments. Perhaps the Finnish DMOs should consider making more efforts in these areas on Chinese social media to increase Chinese tourists’ familiarity with Finnish destinations. Finally, overall, Chinese tourists perceive Finland as “a cultural, history, art, and leisure destination”, which differs from the Finnish DMOs’ promotion of “a nature destination” (VisitFinland 2019). Finnish DMOs, therefore, need to interview Chinese tourists more comprehensively to identify why Chinese tourists do not mention nature-based attributes in their reviews. Are they not interested in nature? Do they fail to access it when they visit? Are they unaware of Finland’s natural attractions? It may be that Chinese tourists prefer to stay in cities and do not venture into nature tourism areas. Thus, the Chinese market might not be the right market to position Finland as a nature destination.

## Conclusion and limitations

In the new era, big data provides a new stream for tourism research (Lu and Stepchenkova 2015). Many earlier studies adopted a single platform as a data source, but they ignored that using single data sources may induce a sampling bias that potentially complicates the interpretation of the research findings. Therefore, in this study, we explored Chinese outbound tourists’ perception of Finland, and compared the discrepancies and commonality of Finland’s image between different OTR platforms. An analysis of Simplified Chinese OTRs proved that different Chinese OTR platforms had a DI discrepancy. The results showed that all nine destination attribute dimensions could be identified in the Chinese perception of the destination. However, when the destination attributes of each dimension were examined more closely, major differences in the destination attributes could be observed on the OTR platforms. In this study, the DI based on different OTR platform content revealed discrepancies at the functional and mixed functional-psychological levels. These differences may be the result of a variety of factors, such as the platform’s target group, market positioning, or other factors. At the psychological destination attribute level, different OTR platforms showed consistency in their representation of the social image, political and economic, and place atmosphere dimensions.

Additionally, rich OTR content could cover all destination attribute dimensions, but each destination is unique when it comes to the scale of the destination attribute. With the development of information technology, OTR platforms have gradually become an important channel for tourists to obtain destination information (Marine-Roig 2017). Especially in the introduction to the functional and the mixed functional-psychological destination attributes, OTRs provide a more comprehensive understanding of destinations. At the psychological level of destination attributes, although Chinese OTRs show an abundant and unique psychological perception of the place atmosphere attribute, less attention was paid to the political and social environment, such as safety issues. The reason for this result may be related to the advantages of OTRs, in that tourists can assist each other on OTR platforms to reduce the risk of travel (Jacobsen and Munar 2012).

Although this study used mixed methods to conduct a comparative analysis of DI represented by various OTR platforms, it has some limitations that may inspire future research. First, an adapted DI framework was applied in this study, and the Finnish attribute coding manual was defined by tourism researchers proficient in Chinese and Finnish culture. However, there is an inevitable degree of subjectivity during the qualitative content analysis process. In this study, OTRs were crawled from Chinese OTR platforms, ignoring the significance of their Western equivalents. To further understand the discrepancies in the same destination’s representation by different OTR platforms, other Western OTR platforms like TripAdvisor, Yelp, or Google Reviews might also be included in future research. Meanwhile, in this study, the discrepancies in the DI on OTR platforms were discussed based on the basic information of the platforms. In order to explain the results of the discrepancies more accurately, other indicators could be considered in future research, such as the actual market and actual product positioning.

The study’s most important limitation is that the analysis process does not calculate the length of the review text, or split the sentences when analyzing the attributes of the phrases in the text, which is common to computerized methods that clean, debug, and analyze large-scale OTR data. Future research could use detailed computerized methods, such as those proposed by Marine-Roig and Clavé (2016), to compare whether the computerized analytical results are consistent with the results of this study method. It is also possible to apply the compositional data analysis approach (CoDa) (Coenders and Ferrer-Rosell 2020; Lalicic et al. 2021) to analyze multiple destinations’ image, thus verifying whether the reasons for the discrepancies between different platforms are the same for different destinations. Besides, although this study has conducted a detailed analysis of each of the destination attributes of the image, due to word limitations, this paper only shows the discrepancies between the various platforms from the perspective of the main DI dimensions. For future research, given the improved performance of big data analysis software, different analysis methods and software could be used to verify the results of this research, such as performing a quantitative analysis using Python. Alternatively, photos could be considered for use as a data source to verify whether discrepancies exist between various OTR platforms.

### Supplementary Information

Below is the link to the electronic supplementary material.

Supplementary file1 (DOCX 31 KB)

## Appendix 1

See Table 5.
