# Supplementary material for: Comparing online travel review platforms as destination image information agents
Source: Inf Technol Tourism. 2021 Apr 22;23(2):159–87. doi: 10.1007/s40558-021-00201-w (PMC8062146; doi:10.1007/s40558-021-00201-w)
Supplement: Supplementary file 1 — Supplementary file1 (DOCX 31 KB) [file 40558_2021_201_MOESM1_ESM.docx]

**Finnish Destination Attributes Codes and Detail Results**

| Name | Nodes |  | References | | | | | | |
| --- | --- | --- | --- | --- | --- | --- | --- | --- | --- |
| **Image Categories of Finland** |  | | | **Total** | **Qyer** | **Ctrip** | **Qunar** | **Mafengwo** | **Tuniu** |
| Image Dimensions | **Destination attributes/Coding** | | | **14489** | **4961** | **3579** | **2019** | **2493** | **1437** |
| Atmosphere of the Place (18) |  | | | **2789** | **889** | **704** | **408** | **444** | **344** |
| Ancient and historical atmosphere(2) | Ancient, Historical | | | 70 | 15 | 23 | 15 | 12 | 5 |
| Artistic atmosphere(2) | Artistic, Creative | | | 202 | 64 | 72 | 30 | 24 | 12 |
| Attractive or interesting(2) | Attractive, Interesting | | | 166 | 51 | 52 | 18 | 18 | 27 |
| Boring(6) | Boring, Un-amazing, Cheating, Non-inserting, Disappointed, discouraged | | | 214 | 108 | 22 | 17 | 41 | 26 |
| Commercialized(2) | Bustling, Commercialized | | | 121 | 57 | 21 | 8 | 22 | 13 |
| Desolate or depression(5) | Lonely, Desolate, Isolated, Shabby, Depression | | | 21 | 10 | 1 | 1 | 4 | 5 |
| Exotic(5) | Unique, Unusual, Peculiar, Featured, Exotic | | | 343 | 88 | 86 | 51 | 60 | 58 |
| Family-oriented destination(1) | Family-oriented destination | | | 207 | 82 | 52 | 29 | 28 | 16 |
| Fun or enjoyable(6) | Satisfying, Exciting, Wonderful, Fun, Enjoyed, Pleasant | | | 98 | 39 | 24 | 7 | 19 | 9 |
| Harmonious(2) | Power Harmony, Racial Harmony | | | 85 | 31 | 12 | 13 | 11 | 19 |
| Majestic(4) | Majestic, Magnificent, Gorgeous, Imposing | | | 171 | 44 | 44 | 34 | 17 | 32 |
| Place with a famous reputation(1) | World famous attractions (popular discussing attractions online) | | | 229 | 43 | 65 | 44 | 52 | 25 |
| Fairytale or magical(3) | Dreamy, Poetic, Fairytale | | | 135 | 37 | 42 | 22 | 18 | 16 |
| Relaxing(4) | Relaxing, Peaceful, Tranquil, Cozy, Laid-back | | | 187 | 69 | 41 | 30 | 26 | 21 |
| Romantic(1) | Romantic | | | 47 | 14 | 14 | 10 | 3 | 6 |
| Solemn(2) | Sacred, Solemn | | | 120 | 29 | 31 | 25 | 8 | 27 |
| Worthy or meaningful(3) | Destinations with travel value, Meaningful destination, Destination with enlightening | | | 373 | 108 | 102 | 54 | 82 | 27 |
| Culture, History and Art (9) |  | | | **2944** | **759** | **823** | **516** | **487** | **359** |
| Ancient City(1) | Porvoo Old Town, | | | 84 | 17 | 25 | 27 | 14 | 1 |
| Art Attractions(8) | Finnish National Gallery, Museum of Contemporary Art Kiasma, Design Museum Helsinki, Culture House Korundi, Amos Rex, Tampere Art Museum, Helsinki Art Museum, Turku Art Museum | | | 208 | 54 | 74 | 41 | 34 | 5 |
| Castle & Fortress(4) | Suomenlinna, Turku Castle,Olavinlinna, Kastelholm Castle, | | | 528 | 140 | 152 | 80 | 109 | 47 |
| Church(9) | Temppeliaukion Church, Uspenski Cathedral, Helsinki Cathedral, Kamppi Chapel, St. John's Church, Turku Cathedral, Porvoo Cathedral, Rovaniemi Church, Kemi Church | | | 1195 | 233 | 297 | 246 | 163 | 256 |
| Festivals(4) | Vappu Day, Easter, Christmas, Midsummer Festival | | | 93 | 32 | 21 | 15 | 20 | 5 |
| Gastronomy(7) | Glögi, Finnish Cranberry Juice, Sima, Finnish salmon soup, Finnish reindeer steak, Finnish smoked salmon, Finnish fried small fish | | | 174 | 102 | 16 | 12 | 33 | 11 |
| Handcraft(2) | Finnish knives, Antler products | | | 33 | 19 | 4 | 2 | 6 | 2 |
| Museum & monuments(22) | The National Museum of Finland, The Polar Museum, Spy Museum, Finnish Museum of Natural History, Luostarinmäki Handicrafts Museum, Hotel & Restaurant Museum, Lenin Museum, Moom in Museum, The Maritime Museum, Kemi Gemstone Gallery, Alvar Aalto Museum, Kemi Gemstone Gallery, Arktikum, Tram Museum, Siida, Finnish Toy Museum Hevosenkenkä, The Finnish Museum of Photography, Suomenlinna Museum, Sibelius Museum, Museum Centre Vapriikki, Ehrensvärd Museum, Museum of Contemporary Art Kiasma. | | | 584 | 150 | 220 | 87 | 97 | 30 |
| Ruins(1) | Sammallahdenmäki | | | 45 | 12 | 14 | 6 | 11 | 2 |
| General Infrastructure (8) |  | | | **2086** | **977** | **376** | **203** | **281** | **249** |
| Bridge(1) | Love Bridge near Uspenski Church | | | 40 | 18 | 8 | 5 | 6 | 3 |
| Educational facility(2) | University, Library | | | 146 | 35 | 44 | 24 | 30 | 13 |
| Financial service facility (1) | Bank | | | 5 | 2 | 0 | 0 | 1 | 2 |
| Government Places(3) | Prison, parliament building, presidential palace | | | 85 | 20 | 25 | 25 | 11 | 4 |
| National industry(2) | Forestry industry, IT industry | | | 23 | 8 | 8 | 3 | 4 | 0 |
| Public Transportation(7) | Bus & Bus Station, Cruises & Terminals, Cycling of Travel, Flight & Airport, Taxi, Train & Train Station, Walking of Travel | | | 1396 | 744 | 214 | 106 | 161 | 171 |
| Shopping & Market Facility(5) | Supermarkets, shopping malls, outdoor free markets, farmers markets, commercial pedestrian streets, | | | 347 | 138 | 64 | 29 | 64 | 52 |
| Street & Road(3) | Road material, road design, road condition | | | 44 | 12 | 13 | 11 | 4 | 4 |
| Natural Environment (4) |  | | | **825** | **257** | **232** | **112** | **126** | **98** |
| Beauty of the scenery(2) | Havis Amanda, Statue of Mannerheim | | | 321 | 72 | 120 | 53 | 39 | 37 |
| Crowded & less of tourists(2) | More tourists, less tourists | | | 408 | 151 | 93 | 47 | 78 | 39 |
| Hygiene Situation(2) | Public waste issues, toilet issues | | | 89 | 30 | 17 | 12 | 9 | 21 |
| Noise pollution(1) | Noisy in the attractions | | | 7 | 4 | 2 | 0 | 0 | 1 |
| Natural Resources (6) |  | | | **800** | **320** | **213** | **94** | **112** | **61** |
| Air(1) | air quality | | | 69 | 13 | 25 | 16 | 8 | 7 |
| Natural Ecosystems(5) | Bay, river, lake, forest, hill | | | 110 | 33 | 54 | 11 | 9 | 3 |
| Natural Phenomenon(1) | aurora | | | 73 | 28 | 25 | 6 | 14 | 0 |
| Season(4) | Spring, winter, autumn, summer | | | 241 | 114 | 48 | 32 | 26 | 21 |
| Variety and uniqueness of flora and fauna(2) | Polar bear, Elk | | | 100 | 25 | 34 | 11 | 21 | 9 |
| Weather(6) | Strong wind, heavy snow, cold climate, sunny, cloudy, rainy, | | | 207 | 107 | 27 | 18 | 34 | 21 |
| Political and Economic Factors (4) |  | | | **511** | **234** | **93** | **59** | **103** | **22** |
| Political Environment(1) | Female congressman | | | 28 | 8 | 11 | 7 | 2 | 0 |
| Political stability(1) | Social harmony and stability | | | 42 | 11 | 14 | 8 | 3 | 6 |
| Prices(6) | Food prices, transportation prices, attraction ticket prices, commodity prices, entertainment prices, accommodation prices | | | 428 | 212 | 64 | 39 | 97 | 16 |
| Safety(3) | Security facilities, police actions, ambulance | | | 13 | 3 | 4 | 5 | 1 | 0 |
| Social Environment (7) |  | | | **340** | **120** | **85** | **42** | **67** | **26** |
| Ethnic origin(2) | the origin of race, and language system, | | | 19 | 3 | 6 | 4 | 2 | 4 |
| Hospitality of local resident(2) | Active knowledge transfer, passionate conversation | | | 44 | 20 | 9 | 6 | 8 | 1 |
| Language barriers(1) | English skill | | | 45 | 24 | 5 | 2 | 12 | 2 |
| Local lifestyle(3) | Local purchases, local education, local lifestyle | | | 140 | 37 | 42 | 14 | 31 | 16 |
| Local values(3) | Advocating nature, animal protection, gender equality | | | 65 | 17 | 23 | 15 | 9 | 1 |
| Underprivileged and poverty(1) | beggar | | | 27 | 19 | 0 | 1 | 5 | 2 |
| Tourist Infrastructure (7) |  | | | **1367** | **651** | **226** | **158** | **251** | **81** |
| Bar(3) | Bar features, bar location, bar drinks | | | 52 | 26 | 11 | 6 | 6 | 3 |
| Destination Accessibility(5) | The sign is not clear, the traffic is convenient, the traffic is inconvenient, the distance is short, the distance is long | | | 505 | 238 | 93 | 61 | 69 | 44 |
| Hotel(5) | Hotel room decoration, hotel service, hotel price, hotel facilities, hotel location, hotel popularity | | | 164 | 69 | 34 | 37 | 19 | 5 |
| Resorts(3) | Resort services, resort scale, resort facilities | | | 23 | 14 | 1 | 2 | 6 | 0 |
| Restaurants(3) | Restaurant dishes, restaurant services, restaurant prices, restaurant decoration | | | 255 | 150 | 28 | 23 | 43 | 11 |
| Tourist Centres(3) | Shopping information service, attraction information service, basic tourist service | | | 247 | 83 | 52 | 17 | 84 | 11 |
| Payment Method(3) | Credit card, cash, Alipay | | | 56 | 28 | 6 | 4 | 13 | 5 |
| Website Information & Services(3) | Official website information query, official website booking service, webcast | | | 65 | 43 | 1 | 8 | 11 | 2 |
| Tourist Leisure and Recreation (10) |  | | | **2827** | **754** | **827** | **427** | **622** | **197** |
| City Park(4) | Esplanadi, Seurasaari, Kaisaniemi park, Sibelius Park | | | 469 | 72 | 145 | 101 | 66 | 85 |
| International events(3) | Ski World Championship, World Athletics Championship, Finnish National Hockey Championship | | | 74 | 35 | 15 | 4 | 18 | 2 |
| National Park(4) | Nuuksio National Park, Oulanka National Park, Koli National Park, Pyhä-Luosto National Park, | | | 280 | 58 | 108 | 50 | 37 | 27 |
| Performances Events(3) | Cabaret, musical instrument, folk performance | | | 85 | 24 | 25 | 12 | 12 | 12 |
| Playground(1) | Särkänniemi playground, | | | 18 | 8 | 5 | 1 | 3 | 1 |
| Sport Activity(4) | Skiing, snowmobiling, snowshoeing, swimming | | | 84 | 34 | 14 | 10 | 23 | 3 |
| Sports Stadium(2) | Helsinki Olympic Stadium, Ounasvaara | | | 108 | 38 | 27 | 7 | 31 | 5 |
| Theme Park(3) | The Snow Castle of Kemi, Angry Birds park, Santa Claus Village, | | | 869 | 251 | 252 | 133 | 201 | 32 |
| Tourist Entertainment(9) | Pick mushrooms, send postcards, sit and relax, watch videos, take pictures, pray, sauna, ride on icebreakers, ride reindeer & huskies | | | 791 | 217 | 215 | 105 | 224 | 30 |
| Zoo & Botanical Garden(3) | Korkeasaari, Ranua Zoo, Kaisaniemi Botanical Gardens | | | 49 | 17 | 21 | 4 | 7 | 0 |
